# Comparative effect of artemether-lumefantrine and artesunate-amodiaquine on gametocyte clearance in children with uncomplicated *Plasmodium falciparum* malaria in Madagascar

**DOI:** 10.1186/s12936-022-04369-2

**Published:** 2022-11-14

**Authors:** Malalanandrianina A. Rakotoarisoa, Jocia Fenomanana, Bronislaw Tchesterico Dodoson, Voahangy Hanitriniaina I. Andrianaranjaka, Arsène Ratsimbasoa

**Affiliations:** 1 Faculté de Médecine, Université d’Antananarivo, Fianarantsoa, Madagascar; 2 Faculté de Médecine, Université de Fianarantsoa, Fianarantsoa, Madagascar; 3 Faculté Des Sciences, Université d’Antananarivo, Fianarantsoa, Madagascar

**Keywords:** Gametocytes, Madagascar, Microscopy, *Plasmodium falciparum*, Artemisinin-based antimalarials

## Abstract

**Background:**

Gametocytes are the sexual stages ensuring continuity of the development cycle of the parasite, as well as its transmission to humans. The efficacy of artemisinin-based anti-malarials against asexual stages of *Plasmodium* has been reported in Madagascar, but their effects on gametocytes are not well documented. The present study aims to determine the emergence of gametocyte and gametocyte clearance after artesunate-amodiaquine (ASAQ) or artemether-lumefantrine (AL) treatment in children with uncomplicated *Plasmodium falciparum* malaria in 5 regions of Madagascar.

**Methods:**

558 children with uncomplicated *P. falciparum* malaria, aged between 1 and 15 years, were assigned randomly to AL or ASAQ treatment. They come from 5 regions of Madagascar with different epidemiological facies related to malaria: Ankilivalo, Benenitra, Ampanihy, Ankazomborona and Matanga. Gametocytes were identified by microscopy, from t blood smears at day 1, day 2, day 3, day 7, day 14, day 21 and day 28 after treatment.

**Results:**

At baseline, 9.7% (54/558) children [95% CI: 7.4–12.5%] had detectable gametocyte by microscopy. Among the 54 enrolled children, gametocytes emergence rate was high during the first days of treatment in both treatment arms (AL and ASAQ), especially on day 1. Gametocytes were undetectable from day 14 for AL arm while for ASAQ arm, gametocyte carriage was gradually decreased but persisted until day 21.

**Conclusion:**

This study demonstrates that AL has a more rapid effect on gametocyte clearance compared to ASAQ in children with uncomplicated *Plasmodium falciparum* malaria.

**Supplementary Information:**

The online version contains supplementary material available at 10.1186/s12936-022-04369-2.

## Background

Although recent studies suggest that the rate of morbidity and mortality linked to malaria has decreased significantly since the use of artemisinin-based combination therapy, this disease remains a major public health problem in many regions of the world [1]. The persistence of transmission depends on the presence of gametocytes in human peripheral blood, which could be ingested by the mosquito during its blood meal. It has been shown that *Plasmodium falciparum* gametocytes are relatively insensitive to several anti-malarials [1–3] and circulate for a longer period than gametocytes from other species [4]. Integrating strategies that target gametocytes into disease control programmes is crucial for controlling or eliminating this disease [5].

According to the World Health Orgnaization (WHO) recommendation, artemisinin-based combination therapy (ACT) is used as first-line treatment for uncomplicated malaria [6]. Indeed, unlike most schizonticides anti-malarials, artemisinin derivatives are effective against the immature stages of gametocytes [7]. However, gametocytocidal effects vary depending on drug regimen, doses administered, local prevalence of anti-malarial drug resistance, host immunity and infectivity of gametocytes [8].

Thus, in Madagascar, the artesunate-amodiaquine (ASAQ) combination is recommended for the first-line treatment of malaria and the artemether-lumefantrine (AL) combination for the second-line according to the national programme for malaria [6]. The efficacy of ACT against asexual stages of plasmodia has been reported in Madagascar [9], but its effect on gametocytes have not been well documented.

The present study aims to determine the emergence of gametocyte and gametocyte clearance after ASAQ or AL treatment in children with uncomplicated *P. falciparum* malaria.

## Methods

### Study sites and sample collection

**Fig. 1:**
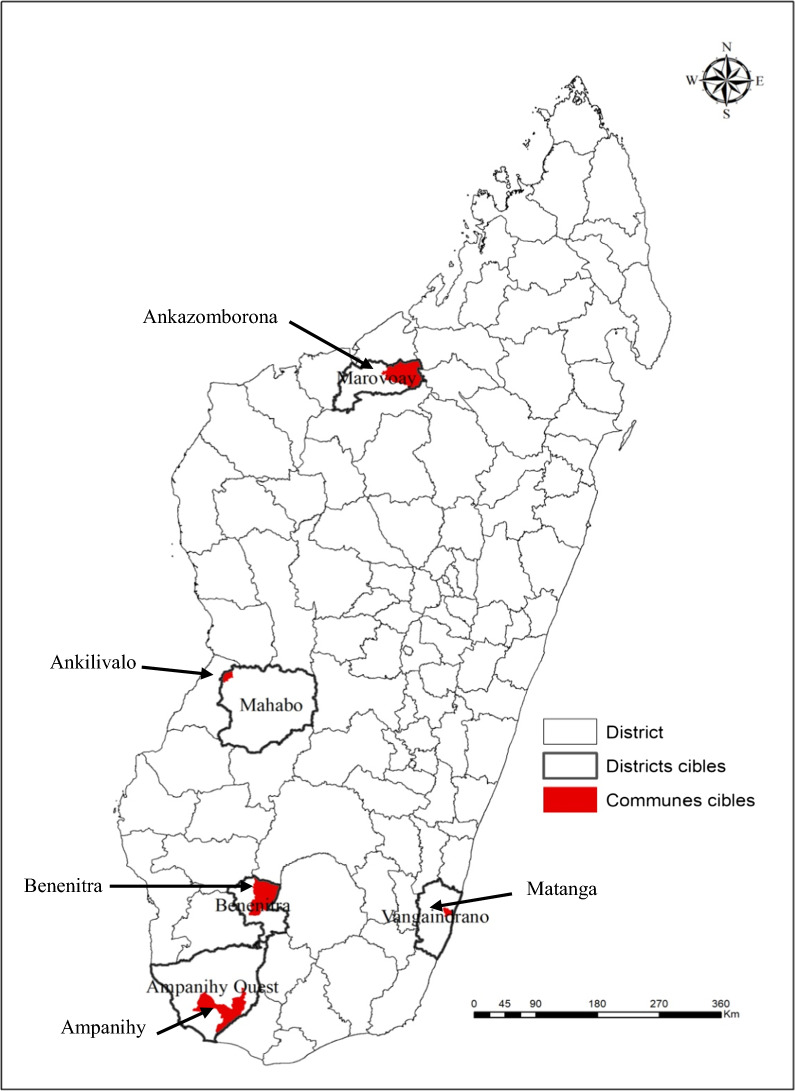
Geographical situation of study sites (Source: Geographical and Hydrographic Institute of Madagascar)

The study was conducted on May 2018 in five sites with different epidemiological facies related to malaria (Fig. 1): Ankazomborona, a district region of Marovoay, located on the west coast of Madagascar (16° 07′ 00″ S and 46° 45′ 00″ E), which is a tropical stratum area with a seasonal, endemic and low transmission, average malaria prevalence of 15.82%. Matanga, a district region of Vangaindrano, located on the east coast of Madagascar (23° 31′ 00″ S and 47° 33′ 00″ E), is an endemic area with stable transmission, average malaria prevalence of 49.78%. Ankilivalo, a district region of Menabe, located on the east coast of Madagascar (20°17′00’’ and 40° 38′00’’), is an area with seasonal transmission during the rainy season with an average malaria prevalence of 11.53%. Ampanihy, a district region of Atsimo-Andrefana, located on the south of Madagascar (24°41′46’’ S and 44°44′46’’ E), is a semi-desert area with an average malaria prevalence of 4.04%. Benenitra, a district region of Atsimo-Andrefana, located on the south of Madagascar (23°26′54’’S and 45°04′46’’E) a semi-desert area, the average prevalence of malaria is 27.60%.

### Sample collection

Samples were collected, from February to April 2018, during an evaluation of the therapeutic efficacy of artemisinin-based anti-malarials, recommended by the Ministry of Public Health of Madagascar for the treatment of uncomplicated malaria in Madagascar.

Two anti-malarials were evaluated:—Artesunate-Amodiaquine Winthrop^®^ (ASAQ), a fixed combination of artesunate-amodiaquine, oral administration, once a day for 3 days. (Additional file 1: Appendix I). Artemether-Lumefantrine Coartem^®^ (AL), a fixed combination of artemether-lumefantrine, oral administration, twice a day for 3 days (Additional file 2: Appendix II).

Initially, microscopic detection of gametocytes was performed on 558 blood smears (using GIEMSA, × 100 magnification, immersion oil) from children with positive *Plasmodium* RDT (SD BIOLINE Malaria Ag P.f/Pan, 05FK63). (day 0, before treatment administration). The microscopic search for gametocyte was repeated on day 1, day 2, day 3, day 7, day 14, day 21 and day 28 after treatment to detect the emergence of gametocytes and to determine the elimination time of gametocytes.

### Slide reading and quality control

An internal and external quality control system was performed before the start of the study. The competence of microscopists to detect, identify and quantify gametocytes has been verified (accreditation) by testing them with a set of slides conforming to the minimum standards described according to the WHO recommendation.

Quantification of gametocytes in thick films was completed against both 500 and 1,000 leukocytes, assuming a leukocyte count of 6,000/µL blood. Each smears was read by 2 microscopists, if any discrepancy was observed between the two results (> 20%), a third reading was performed by a third microscopist. Gametocyte clearance time was measured as the interval between the first and last positive gametocyte smears.

## Results

### Prevalence of gametocytes carriers

In total, 9.7% (54/558) children [95% CI 7.4–12.5%] had detectable gametocyte by microscopy (Table 1) (day 0). The proportion of carriers of gametocytes seen according to the study sites (Table 2) showed no significant difference (p-value = 0.2036).

**Table 1:** Patients characteristics (J0)

	AL	ASAQ	Total
Frequency N	16	38	54
Male (%) (n/N)	56,3 (9/16)	71,1 (27/38)	66,7 (36/54)
Mean age ± ecartype (years)	5,6 ± 3,7 (2–15)	6,4 ± 3,7 (1–15)	6,2 ± 3,7 (1–15)
Average haemoglobin level ± ecartype (g/dl) (Extreme)	9,7 ± 1,2 (8,0–11,7)	9,8 ± 1,7 (8,1–13,9)	9,7 ± 1,5 (8,0–13,9)
Mean temperature ± ecartype (°C)	37,5 ± 1,1 (36,0–39,6)	37,6 ± 1,3 (36,0–41,6)	37,6 ± 1,2 (36,0–41,6)
Mean parasitaemia (parasites/µl)	17 655 (1 284–60 692)	13 093 (6–95 111)	14 445 (6–95 111)

**Table 2:** Proportion of gametocyte carriers by site

Site	Frequency	Percentage %	[IC à 95%]
Ampanihy	10/78	12,8	[6,7–22,8%]
Ankazomborona	12/173	6,9	[3,8–12,1%]
Ankilivalo	3/63	4,8	[1,2–14,2%]
Benenitra	7/71	9,9	[4,4–19,8%]
Matanga	22/173	12,7	[8,3–18,8%]
Total	54/558	9,7	[7,4–12,5%]

A total of 432 blood smear samples (D0, D1, D2, D3, D7, D14, D21 and D28) from the 54 patients with gametocytes were analysed. The patients are all children between 1 and 15 years of age.

### Carriage of gametocytes according to parasitaemia at day 0

**Fig. 2:**
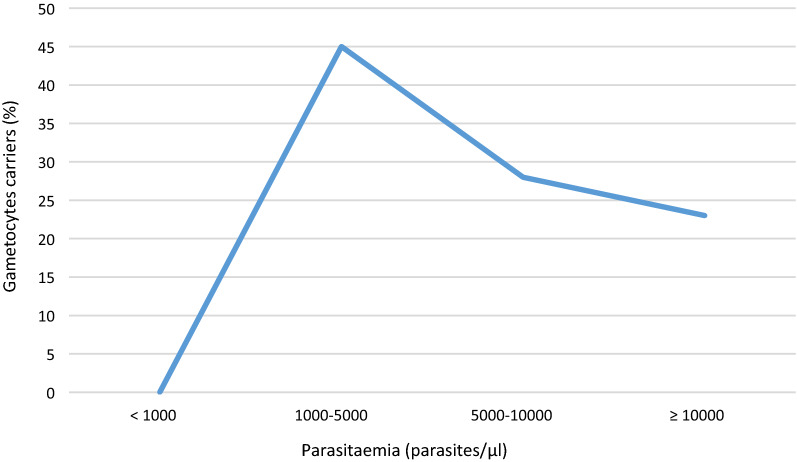
Relationship between parasitaemia and gametocyte carriage at day 0

**Fig. 3:**
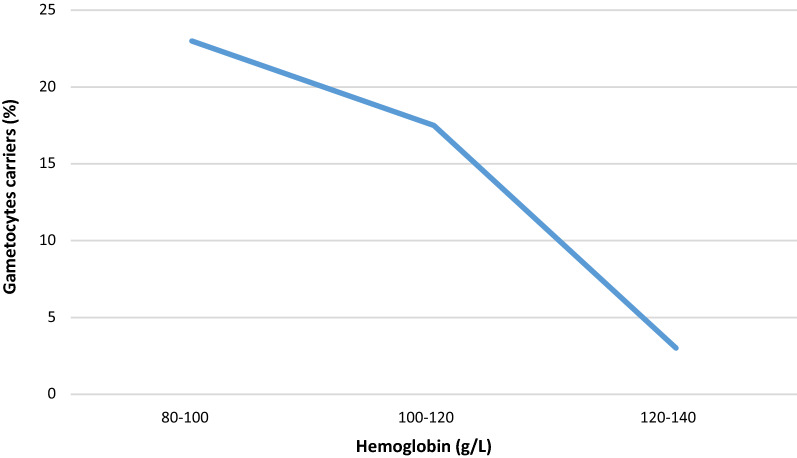
Gametocytes carriers according to the haemoglobin level

The figure below shows the distribution of gametocyte carriage according to parasitaemia on day 0 (Fig. 2). The curve shows a peak of gametocyte carriers for the parasitaemia at the level of 1000 to 5000 parasites/µL. However, no significant relationship between parasitaemia and gametocyte carriage was observed. The carriage of gametocytes is negatively associated with the value of haemoglobin at day 0 (Fig. 3).

### Gametocyte emergence

**Fig. 4:**
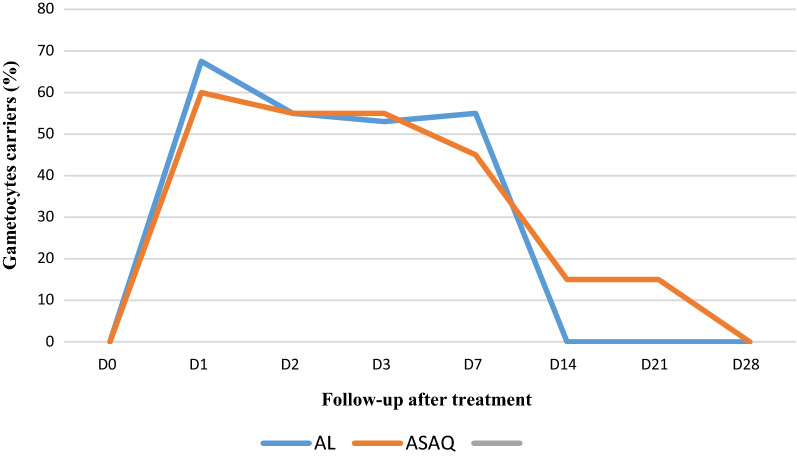
Emergence of gametocytes

Comparing the ASAQ and AL arm, the blood smears collected during the follow-up days (day 1, day 2, day 3, day 7, day 14, day 21, day 28) showed no significant emergence of gametocytes during and after the treatment (Fig. 4).

### Clearance of gametocyte

**Fig. 5:**
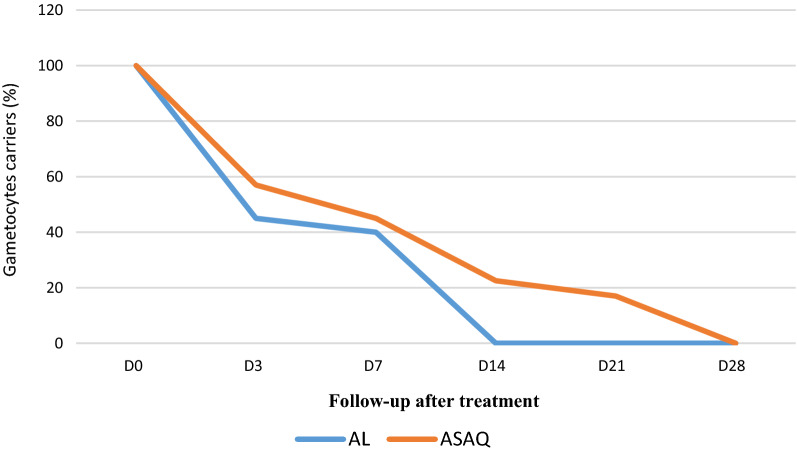
Gametocyte clearance after treatment

The following figure shows the gametocyte clearance curve after treatment (Fig. 5). For the AL arm, the gametocytes are undetectable from day 14 while for the ASAQ arm, the carriage gradually decreases but the gametocytes do not completely disappear until day 28.

(p value: 0.0203). Regarding the overall treatment outcome, the recovery rate following the 2 arms of treatment was 100%.

## Discussion

The carriage of gametocytes is essential for the transmission of *Plasmodium* species to mosquitoes. Understanding the factors influencing gametocytaemia before treatment and the gametocytocidal properties of anti-malarials is of great importance for the implementation of interventions aimed at reducing the transmission of plasmodial infection. According to the literature, gametocytogenesis is sometimes described as a stress response of the parasite which allows it to escape from an increasingly unfavorable environment [10].

The results indicate that the carriage of gametocytes is negatively associated with the value of haemoglobin at day 0. Studies in Thailand and The Gambia found that haemoglobin concentrations were lower in gametocyte carriers [11, 12] and negatively correlated with the number of carriers and the duration of gametocyte carriage [13]. It has been shown that anaemia can also be an independent predictor of gametocytaemia [12] because low haemoglobin concentrations and reticulocytosis directly stimulate the production of gametocytes [14, 15]. The proportion of gametocyte carriers in the population has also been shown to be associated with seasonal fluctuations in the prevalence of anaemia [11].

### Emergence and clearance of gametocytes

It was observed no significant emergence of gametocytes between treatment arms. Studies report that gametocytes found in peripheral blood emerge within the first week of starting treatment [16, 17]. However, the precise mechanism of gametocytogenesis remains unknown [18, 19], but several factors influencing the emergence of gametocytes have been identified, namely genetic factors of the parasite, stresses on the parasites, the exposure to anti-malarials, host immunological factors, mosquito gut microbiota, and even seasonal variation [11–20] or alter gametocytogenesis and, to some extent, affect sexual reproduction in the vector mosquito [21].

Regarding the clearance of gametocytes, the results show that the gametocytes were undetectable from day14 for the AL arm while the carriage gradually decreases but the gametocytes do not completely disappear until day 28 for the ASAQ arm. These results are consistent with previous studies [22]. It has been found that the prevalence of gametocyte carriers after AL treatment decreases steadily from day 0 to day 28, while it increases significantly afterwards for ASAQ [8]. Omondi and his teams have shown that there is no significant difference for the clearance of gametocytes between treatment with LA and with dihydroartemisinin-piperaquine [1].

## Conclusion

This study demonstrates that AL has a more rapid effect on gametocyte clearance compared to ASAQ in children with uncomplicated *Plasmodium falciparum* malaria.

These results will be useful for adjusting policy treatment and orienting strategies to combat malaria in Madagascar.

## Supplementary Information

**Additional file 1: **ASAQ dosage.

**Additional file 2: **AL dosage.

## Data Availability

The data are available from the National Malaria Control Programme of Madagascar.
