# Supplementary material for: Comparative effect of artemether-lumefantrine and artesunate-amodiaquine on gametocyte clearance in children with uncomplicated Plasmodium falciparum malaria in Madagascar
Source: Malar J. 2022 Nov 14;21:331. doi: 10.1186/s12936-022-04369-2 (PMC9664793; doi:10.1186/s12936-022-04369-2)
Supplement: Supplementary file 1 — Additional file 1: ASAQ dosage. [file 12936_2022_4369_MOESM1_ESM.docx]

Appendix I : ASAQ dosage

| **Age group/ weight** | Doses administered during treatment days | | |
| --- | --- | --- | --- |
|  | **1^ère^** | **2^ème^** | **3^ème^** |
| 2 – 11 month*  5 <9 Kg | AS : 25 mg ●  AQ : 67,5 mg | AS : 25 mg ●  AQ : 67,5 mg | AS : 25 mg ●  AQ : 67,5 mg |
| 1 – 5 years  9 – <18Kg | AS : 50 mg ●  AQ : 135 mg | AS : 50 mg ●  AQ : 135 mg | AS : 50 mg ●  AQ : 135 mg |
| 6 – 13 years*  18–<36 Kg | AS : 100 mg ●  AQ : 270 mg | AS : 100 mg ●  AQ : 270 mg | AS : 100 mg ●  AQ : 270 mg |
| > 14 years *  >36 Kg | AS : 100 mg ●●  AQ : 270 mg | AS : 100 mg ●●  AQ : 270 mg | AS : 100 mg ●●  AQ : 270 mg |

**if a discordance between the weight and the age group is observed, the drug is administered according to the weight.*
