# Supplementary material for: Comparative effect of artemether-lumefantrine and artesunate-amodiaquine on gametocyte clearance in children with uncomplicated Plasmodium falciparum malaria in Madagascar
Source: Malar J. 2022 Nov 14;21:331. doi: 10.1186/s12936-022-04369-2 (PMC9664793; doi:10.1186/s12936-022-04369-2)
Supplement: Supplementary file 2 — Additional file 2: AL dosage. [file 12936_2022_4369_MOESM2_ESM.docx]

Appendix II: AL dosage

|  | Doses administered during treatment days | | | | | |
| --- | --- | --- | --- | --- | --- | --- |
| **Weight** | **Day 0** | **Day 0+**  **8 hours** | **Day 1** | **Day 1+12 hours** | **Day 2** | **Day 2+12 hours** |
|  | 0 h | 8 h | 24 h | 36 h | 48 h | 60 h |
| 5 - <15Kg | 1 tablet ● | 1 tablet● | 1tablet● | 1 tablet ● | 1 tablet ● | 1 tablet ● |
| 15 – <25Kg | 2 tablets  ●● | 2 tablets  ●● | 2 tablets  ●● | 2 tablets  ●● | 2 tablets  ●● | 2 tablets  ●● |
| 25– <35Kg | 3 tablets  ●●● | 3 tablets  ●●● | 3 tablets  ●●● | 3 tablets  ●●● | 3 tablets  ●●● | 3 tablets  ●●● |
| ≥ 35 Kg | 4 tablets  ●●●● | 4 tablets  ●●●● | 4 tablets  ●●●● | 4 tablets  ●●●● | 4 tablets  ●●●● | 4 tablets  ●●●● |
